# Alpine-ice record of bismuth pollution implies a major role of military use during World War II

**DOI:** 10.1038/s41598-023-28319-3

**Published:** 2023-01-20

**Authors:** Michel Legrand, Joseph R. McConnell, Gilles Bergametti, Susanne Preunkert, Nathan Chellman, Karine Desboeufs, Laurence Lestel, Andreas Plach, Andreas Stohl

**Affiliations:** 1 Institut des Géosciences de l’Environnement (IGE), CNRS, Université Grenoble Alpes, Grenoble, France; 2 Laboratoire Interuniversitaire des Systèmes Atmosphériques, LISA, CNRS, Université de Paris, Université Paris Est Creteil, 75013 Paris, France; 3 Division of Hydrologic Sciences, Desert Research Institute, Reno, NV USA; 4 Sorbonne Université-CNRS-EPHE, Paris, France; 5 Department of Meteorology and Geophysics, University of Vienna, Vienna, Austria

**Keywords:** Environmental sciences, Atmospheric chemistry

## Abstract

Military conflicts result in local environmental damage, but documenting regional and larger scale impacts such as heavy metal pollution has proven elusive. Anthropogenic emissions of bismuth (Bi) include coal burning and various commodity productions but no emission estimates over the past century exist. Here we used Bi measurements in ice cores from the French Alps to show evidence of regional-scale Bi pollution concurrent with the Spanish Civil War and World War II. Tracers of the main sources of Bi emissions measured in the same ice—coal-burning, steel- and aluminum-industry, alloy and other metal processing—indicate a major, previously undocumented additional emissions source that we attribute to military activities between 1935 and 1945 Common Era (CE) in western Europe. These include the use of bismuth for low-melting point alloys for shells, thin-walled aluminum alloy aircraft oil, and munitions.

## Introduction

Previous studies of past and present trace-metal emissions have focused on the most harmful species such as lead (Pb), cadmium, and mercury^1^. Less attention has been paid to bismuth (Bi) because it previously was thought that Bi was less harmful to environmental and human health. Recent studies, however, now recognize that a range of living organisms are affected by increasing Bi exposure. For example, Bi may reduce sperm metabolism and contribute to infertility in men^2^, and it has been shown to have harmful effects on reproduction in the earthworm species *Eisenia andrei*^3^. Furthermore, earthworm activities in soil may increase Bi bioavailability, potentially enhancing the risk of exposure for other soil organisms such as plants via trophic transfer. Very recent studies indicate that high Bi exposure in garden cress seedlings resulted in toxicity symptoms at morphological and genomic levels^4^. Less documented than for humans and animal cells, the toxicological assessment of Bi for plants remains, however, poorly documented. Together these findings indicate that detailed reconstruction of past environmental Bi contamination is important in terms of impacts on organisms that depend not only on concentration in the environment but also exposure duration.

Sources of atmospheric Bi emissions include coal burning, non-ferrous smelters, and production of aluminium (Al), steel and alloys^5^ but are poorly documented. A few ice-core studies have reported Bi increases from pre-industrial (PI) times to present-day that were attributed either to coal burning^6,7^ or other end use processes^8^, but no comparisons between ice-core data, past coal consumption and commodity production statistics have been conducted to confirm (or not) the importance of these different processes on Bi pollution.

Here, we present a record of Bi deposition (1890–2000 CE) obtained in ice cores extracted from the Col du Dôme (CDD) glacier located near the Mont Blanc summit. Our main goal was to examine the relative importance of different anthropogenic emissions in Europe. This was done by comparing the Bi record with those of species emitted by specific anthropogenic processes including fossil fuel burning and production of different commodities. Bi ice-core data also were compared to estimates of past anthropogenic emissions using the state-of-the-art FLEXPART transport and deposition modeling of atmospheric aerosol.

## Results and discussions

### The CDD records of Bi and other pollutants

**Figure 1:**
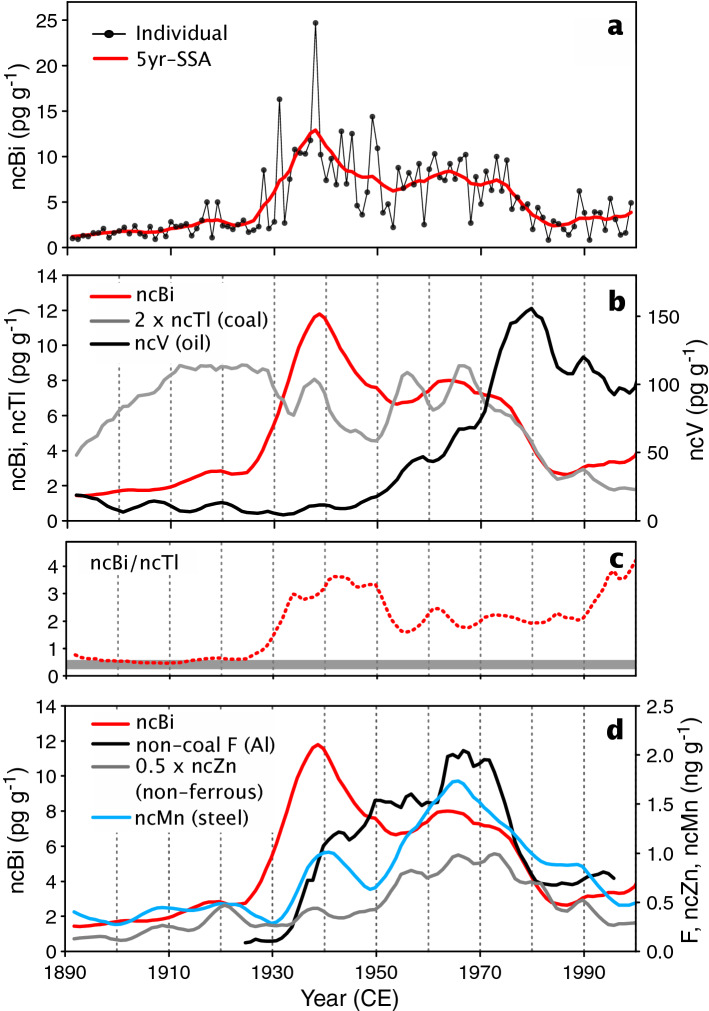
Past concentration changes of non-crustal Bi (ncBi) and other pollutants in summer CDD ice. The red line is the first component of singular spectrum analysis with a 5-yr time window for ncBi (**a**) that is compared in (**b**) to those of ncTl (grey line) and ncV (black line), in (**d**) to non-coal F (black line), ncZn (grey line), and ncMn (blue line). Panel (**c**) shows the ncBi/ncTl ratio in ice (red dashed line), the grey horizontal bar denoting the range of Bi EF-to-Tl EF ratios for coal burning (see “Methods”).

The focus of this study is on long-term changes of non-crustal Bi (ncBi, “Methods”) so we smoothed the ice records (first component of single spectra analysis, SSA, with a 5-yr time window) to minimize the effect of year-to-year variability (Fig. 1a). ncBi concentrations increased slowly from 1890 CE (1 pg g^−1^) to 1925 CE (2.6 pg g^−1^). The ncBi increase accelerated after 1930 CE, with the highest concentrations (higher than 10 pg g^−1^) recorded between 1935 and 1945 CE (Fig. 1a). ncBi concentrations decreased rapidly after 1975 CE but remained three times higher than in 1890 CE. Throughout the twentieth century, ncBi concentrations were well above the PI value (0.27 pg g^−1^, Supplementary Information) indicating that anthropogenic emissions largely dominated natural sources. These changes in ncBi are in agreement with those recorded at Colle Gnifetti (CG, Supplementary Table S1), with both records indicating already high values prior to 1950 CE and a rapid decrease after 1975 CE.

The very different history of ncBi pollution in CDD ice compared to those attributed to fossil fuel burning or commodity manufacturing is illustrated in Fig. 1. The large ncBi increase in the mid-1930s occurred far later that the main 1890–1910 CE increase of non-crustal thallium (ncTl) (Fig. 1b) which has been shown to be a reliable tracer in CDD ice of fallout from coal burning emissions^9,10^. As a result, the ncBi/ncTl ratio (Fig. 1c) remained close to 0.5 (i.e., close to the Bi/Tl ratio in coal burning emissions, “Methods”) but only prior to 1930 CE. In addition, the ncBi increase in the mid-1930s occurred well before the large increase in vanadium (V) fallout in CDD ice previously attributed to increasing oil burning emissions^11^.

**Figure 2:**
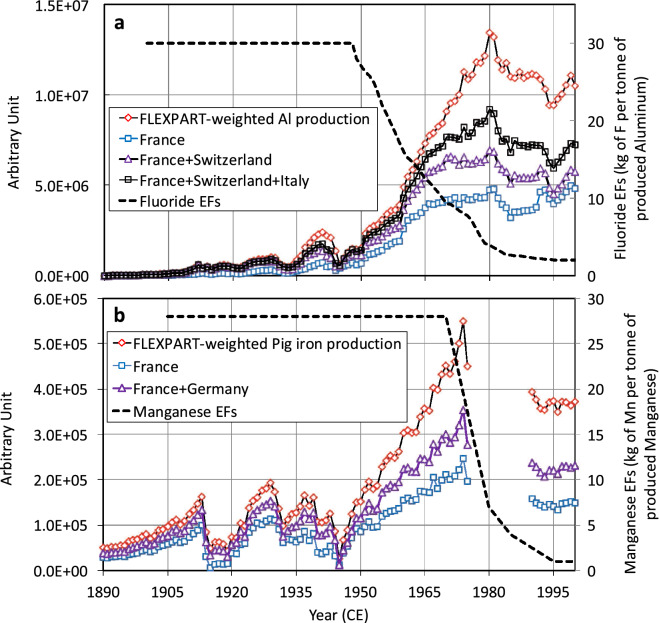
Production of Al (**a**) and pig iron (**b**) from European countries (https://www2.bgs.ac.uk/mineralsuk/statistics/worldArchive.html) scaled by emissions sensitivities estimated by FLEXPART. The dashed black lines are past EFs for F and Mn emitted by Al and pig iron productions, respectively (Table 1).

For emissions related to commodity production, the large increase of the non-coal-burning fraction of fluoride (F) in CDD ice deposited between the late 1930s and 1975 CE (Fig. 1d) previously was attributed to increasing Al production that emitted large amounts of F (30 kg per tonne of Al produced)^12^. The post-1975 CE rapid decrease of F in CDD ice resulted from the reduction of emission factors (EFs) that dropped to 3 kg of F per produced Al tonne in the 1980s (Fig. 2a). The long-term trend of ncZn in CDD ice (Fig. 1d) previously was attributed to non-ferrous smelter emissions that were enhanced particularly after 1950 CE, with the post-1975 CE decrease related to emission reductions^13^.

Past anthropogenic emissions related to pig iron and steel productions have not yet been investigated in CDD ice. European anthropogenic sources of Mn were evaluated for 1979 CE showing main Mn pollution resulted from pig iron and steel production, with West Germany and France being the primary emitters^14^. In 1979 CE, 1200 and 2050 tonnes of Mn were emitted in France and west Germany, respectively, with only ~ 10% resulting from coal burning, 30% from steel production, and 60% for pig iron production. Compared to ncTl, past changes of ncMn concentrations in CDD ice were relatively minor from PI to 1930 CE (Fig. 1), suggesting a weak contribution from coal burning to the anthropogenic Mn budget over Europe. Steel production grew during the twentieth century, reaching a maximum in 1975 CE (Fig. 2b). The CDD ice record is consistent with steel production statistics, with the main ncMn change occurring in the 1950s and 1960s followed by an overall decreasing trend after 1975 CE. The decreasing trend of ncMn concentrations observed in the ice likely was the result of a strong reduction of EFs similar to those seen in Pb emissions from the steel industry (Table 1).

**Table 1:** Estimated emission factors (EFs) of Tl for coal burning, F from Al production, Pb from Pb smelters, and Pb from steel production in Europe from the 1950s to the 1990s and comparison with the amount of Bi that was assumed to be emitted per tonne of consumed (processed) Bi in the 1990s in this study (α, see “Methods”).

	1950	1960	1970	1980	1985	1995	References
EF Tl (coal) (g tonne^−1^)	0.61	0.61	0.44	0.4	0.4	0.2	Ref.^10^
EF F (aluminum) (g tonne^−1^)	27 (13)	16 (8)	9 (4.5)	4 (2)	2.6 (1.3)	2 (1)	Ref.^12^
EF Pb (Pb smelters) (g tonne^−1^)	9000 (4.5)	9000 (4.5)	9000 (4.5)	6000 (3)	3000 (1.5)	2000^a^ (1)	Ref.^33^
EF Pb (iron and steel) (g tonne^−1^)	75 (6.8)	75 (6.8)	75 (6.8)	19 (1.7)	11 (1)	11^a^ (1)	Ref.^33^
α Bi (Bi use) (kg tonne^−1^)	238 (7)	136 (4)	85 (2.5)	68 (2)	51 (1.5)	34 (1)	This work

Values under parenthesis refer to the increase of EF (or α) relative to the 1995 value.

^a^Estimated from ref.^1^.

The Bi trend does not match growing non-ferrous metal production indicated by the CDD ncZn record (Fig. 1d), whereas the large increase of ncBi and ncBi/ncTl in the mid-1930s coincided with a marked increase of non-coal F and ncMn, suggesting that early Bi emissions were related to its use as an additive in aluminum and steel. The most striking feature of the Bi record, however, was the maximum from 1935 to 1945 CE that was well ahead of other pollutants and not attributable to known emission sources.

### Contribution of coal burning, Bi ores, and Pb smelters

**Figure 3:**
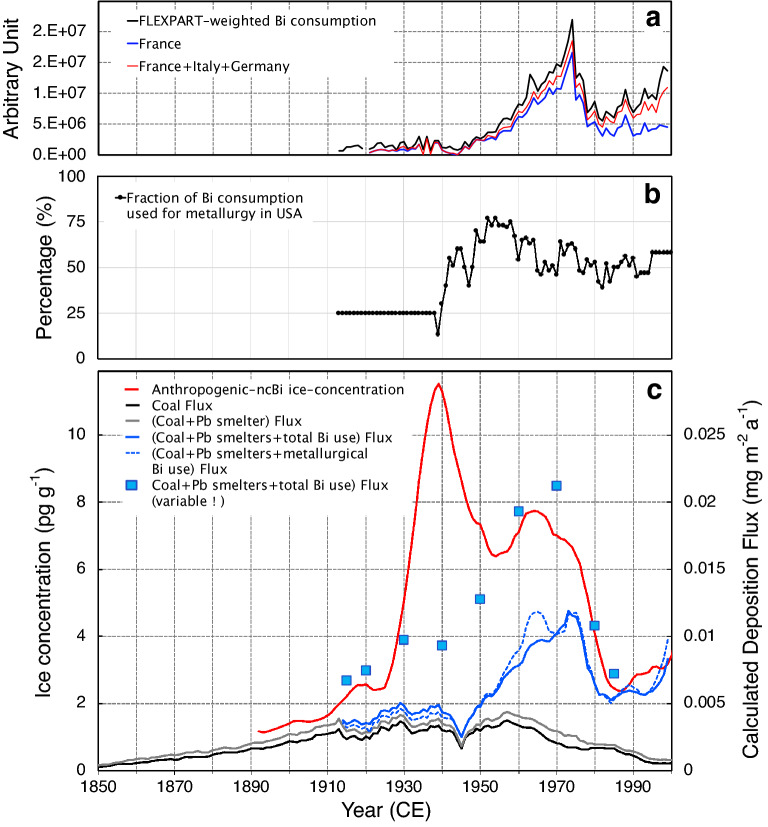
Contribution of coal burning and various metallurgical processes leading to Bi pollution as recorded in CDD ice. (**a**) Bismuth consumption scaled by FLEXPART emission sensitivities of European countries contributing to deposition at CDD. (**b**) Fraction of Bi consumption used for metallurgy in the United States. (**c**) Summer ice-core trends of non-crustal Bi from which the pre-industrial value (0.27 pg g^−1^) was subtracted (red line), and deposition fluxes at CDD calculated by FLEXPART using estimated past anthropogenic emissions from coal burning (black line), coal burning plus Pb smelter (grey line) and coal burning plus Pb smelter and Bi use (blue solid line). The blue squares refer to deposition fluxes calculated by assuming changes over time of the amount of Bi emitted per tonne of Bi consumed (α values, see “Methods”) to account for emission reductions following technological improvements. The dashed blue line refers to emission scenario that considers only Bi use in metallurgy.

Except prior to 1930 CE, Bi deposition fluxes (φ_Bi_) at CDD due to coal burning calculated from the emissions statistics, estimated EFs, and modelled by FLEXPART (see “Methods”) represented less than 20% of total φ_Bi_ (Fig. 3c) for most of the record, confirming the minor contribution of coal burning in the European Bi budget. Based on the high Tl-Bi correlation observed in ice cores extracted in Wyoming^6^, coal burning was suggested to be the main source of Bi pollution there. The Bi/Tl ratio during the pollution maximum at that site was higher than at CDD (8 instead of < 4, Fig. 1c), suggesting either that coal-burning emissions were accompanied by metallurgical emissions or the Bi content of combusted coal was far higher than in western Europe. Examination of coal consumption statistics, Bi content in coal, location of large metallurgical sites and atmospheric transport in North America are needed to understand this difference, but that is outside the scope of this study which is focused on European Bi pollution.

Bi deposition related to the small Bi production from ores in Europe (“Methods”) as calculated by FLEXPART indicate only a relatively small contribution to total Bi deposition at CDD (0.22 10^–3^ mg m^−2^ compared to 1 to 3 10^–3^ mg m^−2^ for coal burning). If present, Bi also will be emitted during Pb ore extraction. Assuming 0.2% Bi in Pb ores (see “Methods”), we calculated that φ_Bi_ from Pb ore extraction was about half of the coal-burning contribution (Fig. 3c). Though an approximation due largely to limited information, these estimates indicate that emissions from coal burning and ore extraction during Bi and Pb production were not large enough to explain the large increase of Bi observed in the ice after 1930 CE.

### Contribution of Bi commodity production and use

From the available statistics, we calculated total Bi consumption for each European country (Fig. 3a). To estimate corresponding φ_Bi_ at CDD using FLEXPART, we assumed that 34 kg of Bi were emitted per tonne of Bi consumed, a value denoted as α_2000_ that was chosen to fit ice-concentrations and FLEXPART deposition fluxes in the late 1990s (Fig. 3c). The α_2000_ of 34 kg of Bi per tonne of Bi consumed is an integrated value that reflects all known production and uses. The true α value probably is lower since the Al industry in western Europe was located mainly near the Alps (France, Italy, and Switzerland, Fig. 2a) where hydroelectricity needed for the electrolytic process was available and CDD would be 3 to 4 times more sensitive to emissions from these nearby alpine regions compared to those from other regions of Italy and France (Supplementary Fig. S4). To a lesser extent, this also applies to steel production with facilities mainly located in eastern France (Alsace, Lorraine) and the Ruhr region of Germany.

In the absence of information on the origin of purified Bi used in pharmacy, we also calculated φ_Bi_ by considering only metallurgical processes instead of total consumption (see “Methods”). Considering that, we determined an α_2000_ value of 70 kg per tonne of Bi used by fitting modelled FLEXPART deposition fluxes in the late 1990s to the CDD measurements. There is, however, no major difference in past φ_Bi_ changes between the two scenarios that considered total or metallurgical Bi uses (Fig. 3c).

The rapid decrease in calculated φ_Bi_ from ~ 1970 to 1990 CE is in line with observed ice-core changes. That reflects a decrease in Bi consumption (Fig. 3c) due to the fact that France—the main European consumer with 1500 tonnes in 1974 CE (67%, Fig. 2a)—drastically reduced its consumption following the 1974 ban of Bi use in pharmaceuticals (https://www.pharmacorama.com/pharmacologie/medicaments-elements/divers/bismuth-pharmacologie/). Prior to 1990 CE, calculated φ_Bi_ values are, however, lower than observed. This likely is due to larger α values at that time compared to 2000 CE. Assuming an α of 1.5 times α_2000_ for 1985 CE, 2.0 times α_2000_ for 1980 CE, 2.5 times α_2000_ for 1970 CE, 4 times α_2000_ for 1960 CE, and 7 times α_2000_ before 1950 CE, results in better agreement from 1950 to 2000 CE between the ice-core trends and FLEXPART-calculated φ_Bi_. This agreement persists back to 1915 CE with the notable exception from 1935 to 1945 CE discussed below. Although the α parameter accounts for emissions from all commodity production (Al, steel, alloys) as well as from commodity uses (e.g., alloys for solders or safety devices), their increases over the past remain quite similar to those of EF values for other industrial processes (Al and steel production, non-ferrous smelters) (Table 1), with relatively minor increases from 2000 to 1980 CE becoming larger back to 1960.

### The large 1935–1945 CE pollution caused by World War II

The largest Bi pollution was recorded in CDD ice between 1935 and 1945 CE. Even if the highest value during the period is eliminated (25 pg g^−1^ in 1938, Fig. 1a), the 1935–1945 peak still reached 10 pg g^−1^. As above discussed, this peak did not coincide with prominent increases of other pollutants (Fig. 1). Furthermore, changes in European Bi consumption and α values adequately explain the ice-core records from 1915 to 1930 CE and 1950–2000 CE (Fig. 3c), but not the peak suggesting additional sources between 1935 and 1945 CE. Use of alloys was the main Bi metallurgical application^15^ at that time and both production and the subsequent use of low-melting-point alloys, may have enhanced emissions compared to years when other applications such as steel additives in the 1960s dominated applications. It is, however, unlikely that α values were enhanced by a factor of 30 (instead of 7 in 1950 CE) with respect to α_2000_, an enhancement that is required to explain concentrations higher than 8 pg g^−1^ seen in ice deposited from 1935 to 1945 CE.

The period between 1935 and 1945 CE coincided with the Spanish civil war (1936–1939 CE) and World War II (1939–1944 CE). Bi demand for alloys during the war was so large that, as stated in the 1939–1945 reports from U.S. Geological Survey statistics (https://www.usgs.gov/centers/nmic/minerals-yearbook-metals-and-minerals), the War Production Board ordered reductions in Bi pharmaceutical use and stockpiling of Bi in national defense industries for thin-walled aluminum alloy aircraft oils. Bi alloys also were used for decompression of gases contained in shells in order to avoid explosion following sudden temperature increases^15^, in addition to plugs for military vehicles to prevent explosion of diesel fuel tanks in fires^16^. Bi also was used in munitions during the two World Wars as a component of chemical irritants (sternutatory)^17^. By their nature, these military uses had higher EFs than civilian uses, resulting in additional Bi emissions.

Considering inherent ice-core dating uncertainties, we cannot rule out that the highest 1938 summer concentration (25 pg g^−1^, Fig. 1a) corresponded either to the so-called “première bataille des Alpes” (June 1940 CE) or the end of the Spanish civil war with the bombing of Guernica and Durango in northern Spain (spring 1937 CE) and Barcelona (March 1938 CE). Note that during World War I (1914–1918 CE), ncBi ice concentrations had already increased from to 2–3 to 5 pg g^−1^ (Fig. 1a). Even higher concentrations also were observed at Colle Gnifetti (CG) between 1910 and 1920 CE (from 6 to 8 pg g^−1^)^7^, although direct comparisons of the summer-only CDD and year-round CG records are of limited value.

## Conclusions

Chemical measurements in alpine ice-cores were used to document Bi pollution in western Europe over the twentieth century. The large increase of Bi concentrations observed in alpine ice layers in the mid-1930s occurred far later that the main 1890–1910 increase of European coal consumption, a finding that calls into question previous assumptions that Bi pollution in this region originated largely from coal burning. We conclude that Bi production and its use in low-melting-point and aluminum alloys, as well as its use as an additive to steel and aluminum, caused most of the Bi pollution in western Europe. Surprisingly, Bi pollution reached its maximum concentration in ice in the years just before and during World War II, probably as a result of military uses including in the aviation industry, in alloys for decompression of gases contained in shells, plugs of vehicles, and in munitions. EF values of these process, however, are virtually unknown, and further studies are needed to better quantify their contribution to past Bi pollution in Europe. We emphasize that the dominance of emissions from production and use of Bi commodities with respect to coal burning would be different in other regions where emissions from metallurgical activities are lower and/or the Bi content in combusted coal is higher.

## Methods

### Ice-core dating and analysis

Chemical analyses were made on ice cores extracted from the CDD site located in the French Alps. Ice cores were dated by annual layer counting primarily using pronounced ammonium seasonal variations (Supplementary Fig. S1), resulting in a composite continuous record from 1890 to 2000 CE (detailed in the Supplementary Information). Because of much greater wind erosion after winter snow deposition, the CDD record better documented summer deposition during the twentieth century (Supplementary Fig. S2). Furthermore, stronger upward transport from polluted boundary layers to high-elevation alpine regions means that pollution reaching CDD is much more directly connected to surrounding surface emissions in summer than in winter. Therefore, we split the chemical records into summer and winter periods and focused discussions on summer trends.

The analyses were conducted using the continuous flow ice core system^18^ at the Desert Research Institute. Longitudinal core samples (3.3 × 3.3 cm) were melted sequentially. Bi and co-analysed cerium (Ce) and manganese (Mn) were measured in meltwater from the innermost 10% of the sample cross-section using two inductively coupled plasma mass spectrometers operating in parallel. Ce was used to estimate the crustal fractions of Bi and Mn (Supplementary Information), the latter used here as a proxy of anthropogenic emissions from the steel industry (Main Text). The detection limits determined as three times the standard deviation of the blank were 0.02 pg g^−1^ for Bi, 0.03 pg g^−1^ for Ce, and 3.0 pg g^−1^ for Mn. As shown in Supplementary Table S1, Bi concentrations in CDD ice were similar to those previously observed at other mid-latitude glacier sites.

### Calculation of non-crustal Bi fraction

Bi is emitted to the atmosphere along with terrigenous particles. With the aim of documenting anthropogenic and natural non-crustal fractions (ncBi), we subtracted from the total measured concentrations the crustal fraction estimated from measured Ce concentrations. Since the Bi-to-Ce ratio in dust reaching CDD can deviate from the “mean sediment” value reported in ref.^19^ we used measurements in crustal-dust-dominated CDD samples to estimate a site-specific Bi-to-Ce ratio (detailed in the Supplementary Information). The observed Bi–Ce relationships in these samples suggested a site-specific crustal Bi/Ce ratio of 0.005 (Supplementary Fig. S3), very close to 0.0048 reported for “mean sediments”^19^. The crustal component of Bi in CDD summer was small, only 7.5% of the total on average.

### FLEXPART model simulations

The Bi trend in CDD ice was used to constrain estimates of past anthropogenic emissions in Europe. To account for effects of atmospheric transport and deposition, we used backward simulations of the Lagrangian particle dispersion model FLEXPART^20^ to determine the sensitivity of the CDD deposition to aerosol emissions in Europe. This approach assumes that variations in ice concentrations and deposition at CDD reflect changes in atmospheric concentrations determined by natural and anthropogenic emissions to the atmosphere. While no information on past precipitation variability at CDD can be derived from ice-core stratigraphy (Supplementary Information), prior studies suggest that precipitation rates in the Alps did not change significantly after 1901 CE^21^ so we assumed that snow deposition conditions at CDD remained similar over the twentieth century.

The model was run at monthly intervals from 1901 to 1999 CE, and particles were traced backward for 30 days. Calculations were made by using the coupled climate reanalysis for the twentieth century (CERA-20C)^22^ performed at the European Centre for Medium Range Weather Forecasts at a 2° × 2° resolution (with 27 vertical layers located below 5000 m asl) every six hours. Supplementary Fig. S4 shows a map of average summer emission sensitivities for the CDD site, with emission sensitivity representing a source-receptor relationship that maps the sensitivity of deposition at the site (receptor) to an emission flux (source). The model was run for 0.4 µm diameter aerosol because metals emitted by anthropogenic sources typically consist of < 1 µm particles^23,24^. Emission sensitivities were averaged for each country to calculate the deposition fluxes at CDD related to European emissions. This was done by weighting emissions from each country by its emission sensitivity and summed.

The FLEXPART-based approach that compares relative changes over the past, however, does not directly assess how well simulated deposition fluxes agree with the ice-core observations. As discussed in ref.^10^, consideration of Pb for which recent past European anthropogenic emissions are much better documented, permitted evaluation of the bias in FLEXPART-simulated deposition largely as a result of the coarse resolution of the model (220 km × 155 km for the CDD grid cell) that poorly resolves alpine orography. Results of this evaluation showed that 3.6 ng g^−1^ of Pb in summer ice would correspond to a summer deposition flux of 9 mg m^−2^ simulated by FLEXPART. Since most metals emitted by anthropogenic activities behave similarly (size distribution, hygroscopicity) with respect to deposition, this relation between Pb ice-concentrations and FLEXPART deposition fluxes at CDD also can be used to evaluate European Bi emissions.

### Anthropogenic processes leading to Bi emissions

Previous studies suggested emissions from coal burning and metallurgical processes were the major sources of anthropogenic Bi pollution. We therefore evaluated the ice-core data initially with respect to these source categories.

For coal burning we used annual coal consumption statistics from ref.^25^ prior to 1975 CE and from British Petroleum^26^ for 1975 to 2000 CE. The coal-burning contribution to Bi deposition (φ_Bi_) at CDD was calculated from estimated EFs (i.e., the amount emitted per mass of burned coal). Unlike toxic metals such as Pb and Tl^27^, coal-burning EFs have not been reported for Bi. Both the abundance in coal and the behavior of Bi during combustion determine the EFs. Large EFs for Bi and Tl in fly ash compared to slag were observed (3 and 2.5, respectively)^24^, suggesting a similar high volatility during combustion. The few published reports of Bi content in coal suggested < 0.05 ppm in most deposits^28^. 0.4 ppm of Tl and 0.2 ppm of Bi were measured in Spanish coal used by a European large power station^24^. Bi was found to be 4 times less abundant than Tl in numerous UK coals^29^. Based on these sparse measurements, we have assumed that the coal-burning EF was three times lower for Bi than for Tl, the latter having been estimated (Table 1) from the examination of past Tl deposition in CDD ice^10^. Here we focused on summertime pollution and we assumed that winter emissions from coal combustion were double those in summer in most European countries^30^. For other emissions sources, it is reasonable to assume no seasonal variability.

There were numerous uses for Bi and its compounds, including in pharmaceutical and cosmetic products, fusible alloys, steel and Al^31^. Metallurgical processes that result in atmospheric Bi emissions include extraction from ores or recovery from lead refining, as well as the production of aluminum, steel, and various alloys. In addition, the use low-melting point alloys for solders resulted in significant Bi releases. Although insignificant atmospheric emissions of Bi compounds would be expected from use in pharmaceuticals and cosmetics, manufacturing them required refined Bi (99.99%) that was produced through thermal processes that emitted Bi. Prior to 1930 CE, Bi was used to treat stomach disorders, wounds, and other ailments, as well as in the manufacture of low-melting-point alloys (containing 50% Bi) used wholly for fire alarms and other safety devices. Bi was used as additive in aluminum (0.2–0.7%) and in the 1950s in steel (0.1–0.5%) to improve machinability.

Bi production from ores in Europe is very limited, with 100–200 tonnes reported between 1920 and 1945 CE in Spain, and 50–100 tonnes from 1945 to 1990 CE at the Salsigne gold mine (France) (https://www2.bgs.ac.uk/mineralsuk/statistics/worldArchive.html). Because of its high volatility, we assumed that EFs were similar for Bi and Pb during ore extraction (i.e., 9 kg per t of Bi produced prior to 1975 CE, Table 1). If present, Bi also will be emitted during Pb ore extraction. The Bi content in Pb ores ranges from zero up to a few %^32^ but no information is available for Pb ores processed in Europe. We calculated φ_Bi_ from Pb ore extraction assuming 0.2% Bi content in ores processed in European Pb smelters (previously estimated in ref.^13^).

Historical statistics on European production of aluminum and steel exist but not detailed information on past changes of the amount of Bi added to Al and steel. Furthermore, no statistics are available on other Bi commodity production such as alloys or on the amount of refined Bi that was produced domestically (and/or imported) for pharmaceuticals and cosmetics in each European country. Given this lack of information, we compared the ice-core record to total Bi consumption derived from available statistics on production, import, and export of Bi (British Geological Survey, https://www2.bgs.ac.uk/mineralsuk/statistics/worldArchive.html), as well as to Bi consumption used for metallurgy (excluding pharmaceuticals). The latter was calculated from the fraction of Bi used during the twentieth century for metallurgy in the United States since such information is not available for Europe (Fig. 3b).

## Supplementary Information

Supplementary Information.

## Data Availability

Ice core data will be available at NCEI (National Centers for Environmental Information) data base (https://www.ncdc.noaa.gov/data-access/paleoclimatology-data) (submitted on 26.12.2022, Reference ID: YJX30L).
