# Supplementary material for: Alpine-ice record of bismuth pollution implies a major role of military use during World War II
Source: Sci Rep. 2023 Jan 20;13:1166. doi: 10.1038/s41598-023-28319-3 (PMC9859787; doi:10.1038/s41598-023-28319-3)
Supplement: Supplementary file 1 — Supplementary Information. [file 41598_2023_28319_MOESM1_ESM.pdf]

Supplementary Information for

Alpine-ice record of bismuth pollution implies a major role of military use during World War II

Michel Legrand[1,2], Joseph R. McConnell[3], Gilles Bergametti[2], Susanne Preunkert[1], Nathan Chellman[3], Karine Desboeufs[2], Laurence Lestel[4], Andreas Plach[5], & Andreas Stohl[5]

This file includes:

Supplementary Text
Text S1. Ice-core Material and Dating
Text S2. The crustal ratios of Bi and Mn deposited in alpine ice
Supplementary Table S1
Supplementary Figs. S1 to S4

Text S1. Ice-core Material and Dating

To investigate long-term trends of pollutants over the 20th century, chemical measurements were made on two ice-cores extracted at the Col du Dome site (45°N, 7°E, 4250 m above sea level) located in the French Alps. The C10 core (126 m long) was drilled in 1994 CE and the CDM core (140 m long) in 2012 CE.

The upper sections of both cores were dated by annual layer counting using well-marked seasonal variations in ammonium concentrations (Supplementary Fig. S1). The resulting C10 chronology was assigned an age of 1890 CE at 118.3 m as detailed in ref.[1], the C10 and CDM records were combined to develop a composite, continuous record from 1890 to 2000 CE. Winter layers generally thin with depth relative to summer layers because of a lack of preservation of blowing winter snow upstream of the CDD drilling site. Although winter layers can be identified back to 1890 CE, for some years the boundary of the winter half-year defined as at least three consecutive samples having ammonium level lower than 10 ng g^{-1} (ref.[2]) is not reached. From 1891 to 1929 CE many of the winter ammonium minima were too thin to determine reliably winter concentrations, and only six winter values were calculated (Supplementary Fig. S2). We emphasize that no information on past precipitation changes at CDD can be derived from the ice-core stratigraphy which only provides annual layer ice thickness and includes effects of wind redistribution (accumulation or erosion).

Prior to 1890 CE, no winter snow was preserved, but the age from 123 to 125 m depth of the C10 core was estimated in ref.[3] to cover time periods beyond the onset of PI (~1850 CE). These summer ice layers were analysed to evaluate the PI values that were of 0.42 ± 0.25 pg g^{-1} (0.27 ± 0.20 pg g^{-1} for ncBi).

Text S2. The crustal ratios of Bi and Mn deposited in alpine ice

As done in previous CDD ice-core studies[3-5], the metal/Ce ratio of crustal aerosol deposited at the CDD site have been estimated by examining the relations between Bi and Ce ice in which the dust contribution dominated the anthropogenic fractions. This was done first in CDD ice samples deposited after 1870 CE containing Saharan dust (Supplementary Fig. S3). These samples are characterized by calcium-rich alkaline layers[6], so we estimated the acidity

(or alkalinity) of samples by checking the ionic balance between anions and cations (e.g., $[H^+]=([Cl^-]+[NO_3^-]+[SO_4^{2-}])-([Na^+]+[Mg^{2+}]+[Ca^{2+}]+[NH_4^+]$, with concentrations expressed in micro-equivalents per liter). Since Ce is present in relatively water-insoluble dust particles, measurements are influenced by acidification times. Previous assessment of measurement recovery with the DRI system for which the on-line acidification during continuous measurements is limited to a few minutes indicated that recovery was 100% and 60% for Pb and Ce, respectively[7]. We assume a similarly high recovery for Bi since it is also mainly associated with pollution in 20th century CDD ice. The presence of Saharan dust significantly enhanced the level of Bi in CDD ice and was large enough with respect to anthropogenic input to estimate the crustal contribution (Supplementary Fig. S3). We also examined the relationship between Bi and Ce in samples containing low dust concentrations. To minimize the contribution of anthropogenic input in these samples, we restricted the examination to samples deposited during PI. Consistent with the first approach, Supplementary Fig. S4b indicated a lower envelope of the correlation between Bi and Ce with a slope of 0.008. Note that the published "mean sediment" Bi/Ce ratio is 0.0048[8] so the same of the CDD-specific ratio of 0.008 when considering the under-recovery of Ce data of 1.7. We used a similar approach to evaluate the CDD-specific ratio of Mn/Ce (Supplementary Fig. S3c,d) to calculate ncMn used to trace back emissions from the steel industry (Supplementary Fig. S3c). This approach leads to a value of 13.3 (8 when considering the under-recovery of Ce data) instead of 9.3 in mean sediment[8].

Supplementary References

1. Legrand, M. et al. Alpine ice evidence of a three-fold increase in atmospheric iodine deposition since 1950 in Europe due to increasing oceanic emissions. *P. Natl. Acad. Sci. USA* **115**, 12136-12141 (2018).

2. Preunkert, S., Wagenbach, D., Legrand, M., & Vincent, C. Col du Dôme (Mt Blanc Massif, French Alps) suitability for ice-core studies in relation with past atmospheric chemistry over Europe. *Tellus B* **52**, 993–1012 (2000).

3. Legrand, M. et al. Cadmium Pollution from Zinc-Smelters up to Fourfold Higher Than Expected in Western Europe in the 1980s as Revealed by Alpine Ice. *Geophys. Res. Lett.* **47**, e2020GL087537 (2020).

4. Legrand, M. et al. Thallium Pollution in Europe over the Twentieth Century Recorded in Alpine Ice: Contributions from Coal Burning and Cement Production. *Geophys. Res. Lett.* **49**, e2022GL098688 (2022).

5. Arienzo, M. et al. Alpine ice-core evidence of a large increase in vanadium and molybdenum pollution in Western Europe during the 20th century. *J. Geophys. Res.- Atmos.* **126**, e2020JD033211 (2021).

6. Wagenbach, D., Preunkert, S., Schaefer, J., Jung, W., & Tomadin, L. in *The impact of African dust across the Mediterranean*, (eds Guerzoni, S. & Chester, R) 291-300 (Kluwer Academic Publishers, 1996).

7. McConnell, J. R. et al. Pervasive Arctic lead pollution suggests substantial growth in medieval silver production modulated by plague, climate, and conflict. *P. Natl. Acad. Sci. USA* **116**, 14910–14915 (2019).

8. Bowen, H. *Trace Elements in Biochemistry* (Academic Press, 1996).

9. Kaspari, S. et al. Recent increases in atmospheric concentrations of Bi, U, Cs, S and Ca from a 350-year Mount Everest ice core record. *J. Geophys. Res.* **114**, D04302 (2009).

10. Gabrielli, P. et al. Early atmospheric contamination on the top of the Himalayas since the onset of the European Industrial Revolution. *P. Natl. Acad. Sci. USA* **117**, 3967-3973 (2020).

11. Chellman, N. et al. Reassessment of the Upper Fremont Glacier Ice-Core Chronologies by Synchronizing of Ice-Core-Water Isotopes to a Nearby Tree-Ring Chronology. *Environ. Sci. Technol.* **51**, 4230-4238 (2017).

12. Sigl, M. et al. 19th century glacier retreat in the Alps preceded the emergence of industrial black carbon deposition on high-alpine glaciers. *The Cryosphere* **12**, 331-3331 (2018).

13. Pisso, I. et al. The Lagrangian particle dispersion model FLEXPART version 10.4. *Geosci. Model Dev.* **12**, 4955–4997 (2019).

Table S1. Bi mean concentrations in the CDD ice (this work) compared to those previously at the non-polar sites of CG in the Alps, UFG in the Wyoming, and in Himalaya. For all sites we separated data covering pre-industrial times (PI) from those corresponding to present-day (PD).

Sites	Elevation	Bi (pg g^{-1})		
		PI	PD maximum	PD recent
Himalaya[a] 28.03°N, 86.96°E	6518 m	2.2	7.1 (1970-2002 CE)	7.1 (1970-2002 CE)
Himalaya[b]	7200 m	1.0	4.1 (1950-1992 CE)	4.1 (1950-1992 CE)
UFG[c] 41.1°N, 109.6°W	4100 m	0.23	8.4 (1890-1930 CE)	1.66 (1960-1984 CE)
CG[d] 45.9°N, 7.8°E	4455 m	1.24	4.4 (1901-1950 CE)	3.6 (1951-1993 CE)
CDD[e] 45.8°N, 6.8°E	4250 m	0.4	6.6 (1935-1975 CE)	3.4 (1980-2000 CE)

[a]Ref.[9], [b]Ref.[10], [c]Ref.[11], [d]Ref.[12], [e]This work

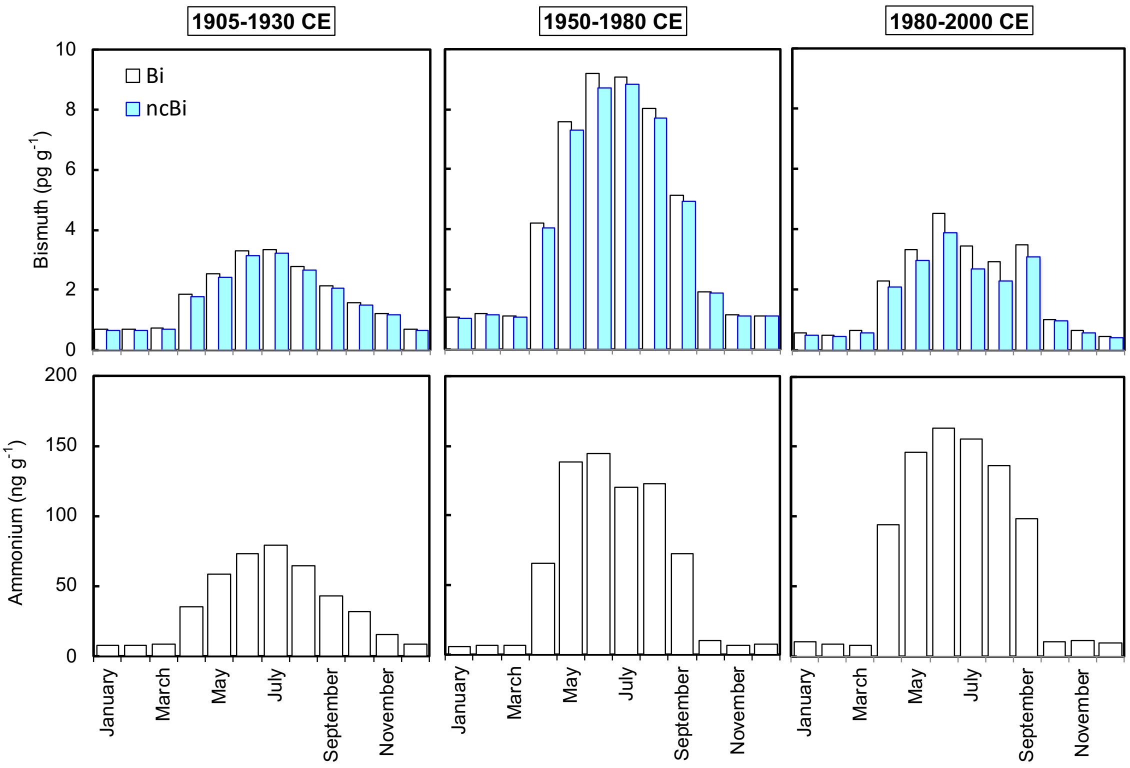

Figure S1. Mean seasonal cycles of bismuth, and of its non-crustal fraction (ncBi, blue vertical rectangles, see Methods), and ammonium (used for seasonal dissection) in CDD ice deposited between 1905 and 1930 CE (left), 1950 and 1980 CE (middle), and 1980-2000 CE (right). Note the change of units from pg g^{-1} for Bi to ng g^{-1} for NH_4^+.

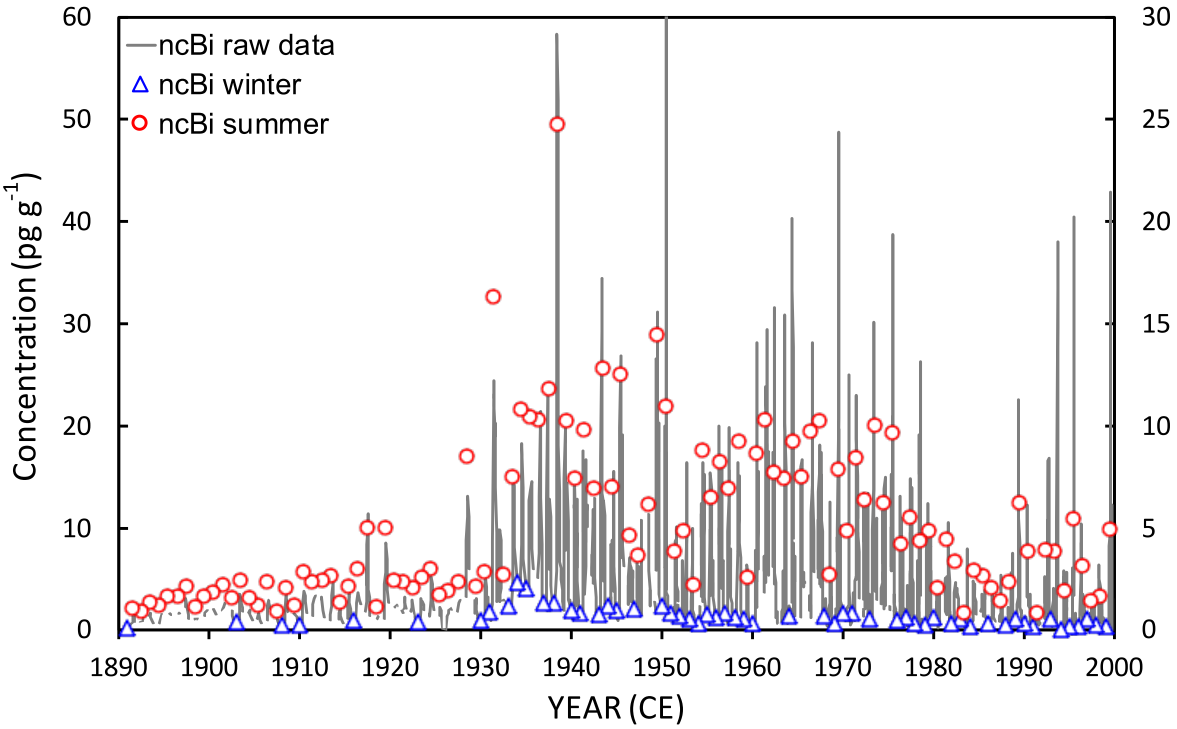

Figure S2. The 1890-2000 CDD ice records of non-crustal bismuth (ncBi). The grey lines refer to raw data (left scale), the red and blue symbols to half-year summer and winter values, respectively (right scale).

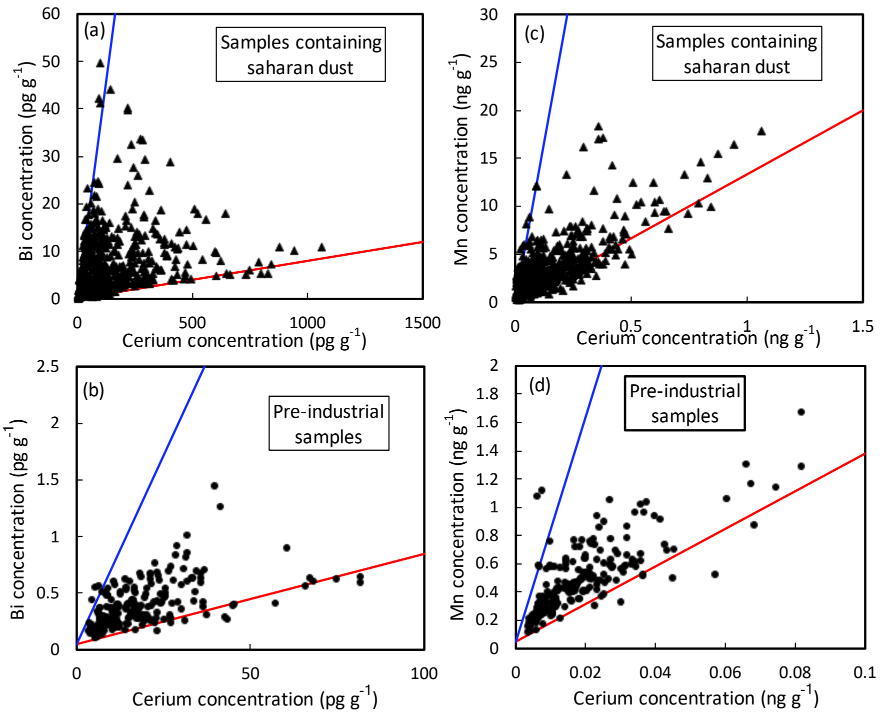

Figure S3. Bi and Mn versus Ce (non-corrected from under-recovery, Text S2) concentrations in CDD samples containing large Saharan dust (a for Bi and c for Mn) or deposited prior to 1870 CE (b for Bi and d for Mn). The red and blue lines illustrate the upper and lower envelopes of the Bi (and Mn)-to-Ce relationships, respectively. Note the change of units from pg g^{-1} for Bi-Ce plots to ng g^{-1} for Mn-Ce ones.

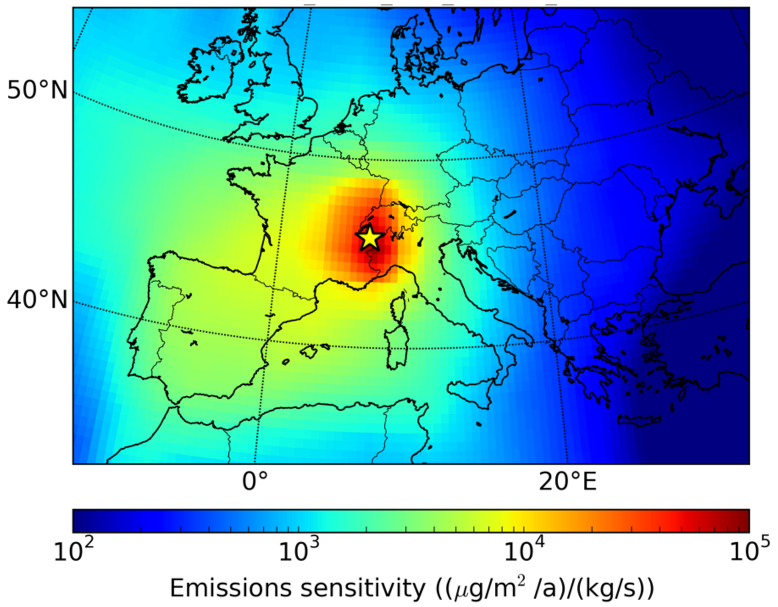

Figure S4. Average summer emissions sensitivities (in (µg m^{-2} a^{-1})/(kg s^{-1})) at the CDD site (yellow star) based on FLEXPART model simulations (version 10.4)[13] for deposition of submicron aerosol.
